# Dispatch and prehospital transport for acute septic patients: an observational study

**DOI:** 10.1186/s13049-017-0393-x

**Published:** 2017-05-12

**Authors:** Peter Bank Pedersen, Daniel Pilsgaard Henriksen, Søren Mikkelsen, Annmarie Touborg Lassen

**Affiliations:** 1 Department of Emergency Medicine, Institute of Clinical Research, University of Southern Denmark & Odense University Hospital, Odense, C DK-5000 Denmark; 2 Department of Emergency Medicine & Department of Respiratory Medicine, Odense University Hospital, Odense, C DK-5000 Denmark; 3 Department of Anesthesiology and Intensive Care Medicine, Odense University Hospital, Odense, C DK-5000 Denmark; 4 Department of Emergency Medicine, Institute of Clinical Research, University of Southern Denmark & Odense University Hospital, Odense, C DK-5000 Denmark

**Keywords:** Prehospital transport, Dispatch, Sepsis, Septic shock, Emergency medicine, Acute medicine

## Abstract

**Background:**

In order to dispatch ambulances with the correct level of urgency, the dispatch center has to balance the perceived urgency and traffic safety considerations with the available resources. As urgency is not clear in all clinical situations, some high urgency patients may end up with a suboptimal mode of transport.

Patients with severe sepsis or septic shock suffer from highly time dependent conditions but they present with a wide range of symptoms, which might be difficult to identify in the dispatch system.

The aim of the study is to investigate the modes of prehospital transport among acute admitted patients with sepsis, severe sepsis and septic shock.

**Methods:**

We included all adult patients (≥15 years) presenting to an acute medical unit at Odense University Hospital with a first-time admission of community-acquired sepsis between September 2010-August 2011. Cases and prehospital ambulance transport were identified by structured manual chart review. In all cases it was registered, whether the ordinary ambulance was assisted by the mobile emergency care unit (MECU), manned by anesthesiologists.

**Results:**

We included 1,713 patients median age 72 years (IQR 57–81), 793 (46.3%) male, 621 (36.3%) had sepsis, 1,071 (62.5%) severe sepsis, and 21 (1.2%) septic shock.

In the group of sepsis patients, 390 (62.8%) arrived without public prehospital transport, 197 (31.7%) were transported by ambulance, and 34 (5.5%) were assisted by MECU. In the group of severe sepsis patients, the same percentage 62.8% arrived without public pre-hospital transport, a lower percentage 28.2% were transported by ambulance, and a larger percentage 9.0% were transported by MECU. Among 21 patients with septic shock, 10 arrived without public pre-hospital transport (47.7%), 7 (33.3%) were transported by ambulance, and 4 (19.0%) by MECU.

The 30-day mortality hazard ratio was associated with mode of transport, with the adjusted highest hazard ratio found in the group of MECU transported patients 1.76 (95%Cl 1.16–2.66).

**Conclusions:**

A substantial proportion of patients with severe sepsis and septic shock arrive to hospital without public prehospital transport or by unspecialized ambulances.

## Background

Emergency medical dispatchers are gatekeepers for pre-hospital emergency care and receive calls from patients or bystanders [1]. Optimal dispatch of prehospital patient transport is a balance between expected urgency, considerations pertaining to traffic safety and available resources. Patients need different levels of transport, from patients with life threatening conditions, which require immediate response by mobile emergency care units (MECU), to patients who attend the hospital without public prehospital transport. The response from the dispatch system is based at descriptions of symptoms and conditions, and aim to identify patients with an acute life-threatening condition, who should receive a more rapid ambulance response, than patients with a non-acute condition [2]. The aim is that the correct mode of transportation is dispatched to all patients at all time [3]. However, urgency is not clear in all clinical situations, and therefore some high urgency patients end up with a suboptimal mode of transport [4].

Patients with severe sepsis, which is a life-threatening organ dysfunction caused by a dysregulated host response to infection, or septic shock, suffers a highly time dependent condition. [5–7]. As septic patients present with a wide range of symptoms, it can be difficult to identify them in the dispatch as well as in the hospital system [5, 8, 9].

The aim of the study is to present the mode of prehospital transport among acute admitted patients with sepsis, severe sepsis and septic shock. We hypothesized that mode of prehospital transport was independent of sepsis severity at arrival to hospital.

## Methods

This study is a hospital based observational study based on registration of all acute medical hospital contacts and MECU transports to the hospital in combination with chart reviews of all acute medical patients. All patients who arrived with sepsis, severe sepsis or septic shock were identified by post-hoc diagnosis. The identification methods have previously been described in details [10–15].

### Setting

The Danish healthcare system is tax-funded and provides free healthcare for all residents. Odense University Hospital serves as a primary hospital for a population of 290.000 persons.

The acute medical unit had all acute admitted medical patients, referred either from a primary care physician or from the open general emergency department. Exceptions were patients with suspected intracranial thrombosis or hemorrhage, with severe cardiac disease, in hemodialysis, patients in chemotherapy or radiation therapy, or women in active labour.

### Patients

We included all adult patients (≥15 years) admitted to an acute medical unit at Odense University Hospital, Denmark, catchment area with maximum distance approximately 40 km, in a 1 year period from September 2010, to August 2011. All contacts were evaluated by a structured manual chart review of the electronic patient file. All patients with sepsis, severe sepsis and septic shock were identified based on predefined criteria of their symptoms at arrival, in combination with results of laboratory findings, cultures and other diagnostics within the first 48 h after arrival [16].

Patients with sepsis of any severity, who died in the open general ED, were included as well. Patients hospitalized up to 7 days before the current admission were not included to exclude possible hospital-acquired infection. Patients transferred from other hospitals, and patients residing outside the hospitals catchment area at the time of admission, were excluded [10].

Using the unique Danish personal identification number [17], supplemental information was retrieved from the Civil Registration System in Denmark [18] and The Danish National Patient Register [19].

Type of prehospital transport was identified by electronic registration of all MECU transports and information linked at personal level by the unique personal identification number. Ambulance transports were registered by an identification paper, filled by the emergency medical technicians. In all cases where there was no identification of prehospital MECU or ambulance transport we performed a structured manual review of the electronic patient files, where we identified any copy of the ambulance file (optional to store) or other registrations regarding mode of transport.

### Definitions

Systemic inflammatory response syndrome (SIRS) definition: At least two of the following criteria: Temperature >38 °C or <36 °C, pulse >90 beats/min, respiratory rate >20 breaths/min or PaCO2 < 4,3kPa (<33 mmHg), leukocyte >12x10mia/L or <4x10mia/L. The vital signs used were baseline measurements.

Sepsis was SIRS plus a documented or suspected infection. Severe sepsis was sepsis and at least one organ dysfunction. Septic shock was the occurrence of sepsis plus a systolic blood pressure ≤ 90 mmHg and a lactate >4,0 mmol/L within 4 h after arrival to the hospital or the use of vasopressor agents within the first 24 h after arrival [10].

### Analysis

Data were presented as proportions with 95% confidence intervals or median and interquartile range as appropriate.

Patients were included at their first visit with severe infection within the period. Patients were followed until death, emigration, or 30 days after admission, whichever came first.

We categorized patients into sepsis, severe sepsis or septic shock and categorized prehospital transport into MECU-assisted transportation or ordinary ambulance. If no prehospital mode of transport could be identified, we categorized the patients as attending the hospital without public prehospital transport.

With the aim to describe an alternative estimate of severity of disease and prehospital transport mode, we presented two Cox proportional hazard regression models where the outcome of interest was all-cause mortality within 30 days mode of transport. We presented two models: (i) A crude analysis, (ii) A multivariable analysis adjusted for age and gender, and a multivariable analysis adjusted for all of the remaining potential risk factors (fully adjusted model), dichotomous variables were set as ‘not present’ or ‘within normal range’ if missing. Statistical analyses were performed with Stata version 14.1 (Stata Corporation LP, TX). The reporting of this study conforms to the reporting of observational studies in epidemiology statement [20].

## Results

We included 1,713 patients with sepsis of any severity, 793 (46.3%) were male. The median age was 72 years (IQR 57-81). The predominant site of infection was the lower respiratory tract (62.9%), and the most prevalent site of organ failure was the lungs.

Among the 1,713 included patients with sepsis at any severity, 621 patients (36.3%) had sepsis, 1,071 patients (62.5%) severe sepsis, and 21 patients (1.2%) septic shock.

Patients who were transported by MECU more often had bacteremia, central nervous infection and central nervous system failure, metabolic failure or coagulation failure as site of organ failure (Table 1).

**Table 1:** Baseline characteristics of sepsis patients arriving without public prehospital transport, ambulance or MECU to an Acute Medical Unit in a 1-year period

Mode of transport		All N	Without public prehospital transport	Ambulance	MECU
Sex	Total	1713	(*n* = 1,073)	(*n* = 506)	(*n* = 134)
	Female	920	601 (56.0%)	251 (49.6%)	68 (50.7%)
	Male	793	472 (44.0%)	255 (50.4%)	66 (49.3%)
Age in age categories, years	15–39	633	437 (40.7%)	153 (30.2%)	43 (32.1%)
	40–64	815	464 (43.2%)	280 (55.3%)	71 (53.0%)
	65–84	265	172 (16.0%)	73 (14.4%)	20 (14.9%)
Charlson Comorbidity Index	0	570	394 (36.7%)	140 (27.7%)	36 (26.9%)
	1	415	240 (22.4%)	136 (26.9%)	39 (29.1%)
	>2	728	439 (40.9%)	230 (45.5%)	59 (44.0%)
Immunosuppression	No	1362	863 (80.4%)	400 (79.1%)	99 (73.9%)
	Yes	351	210 (19.6%)	106 (20.9%)	35 (26.1%)
Sepsis severity	Sepsis	621	390 (36.3%)	197 (38.9%)	34 (25.4%)
	Severe sepsis	1071	673 (62.7%)	302 (59.7%)	96 (71.6%)
	Septic shock	21	10 (0.9%)	7 (1.4%)	4 (3.0%)
Bacteremia	No	1541	967 (90.1%)	460 (90.9%)	114 (85.1%)
	Yes	172	106 (9.9%)	46 (9.1%)	20 (14.9%)
Number of sources of infection per patient	1	1453	910 (84.8%)	428 (84.6%)	115 (85.8%)
	2	242	152 (14.2%)	73 (14.4%)	17 (12.7%)
	3	18	11 (1.0%)	5 (1.0%)	2 (1.5%)
Sites of infection	Lower respiratory tract	1077	636 (59.3%)	339 (67.0%)	102 (76.1%)
	Urinary tract	415	273 (25.4%)	113 (22.3%)	29 (21.6%)
	Abdominal	184	129 (12.0%)	49 (9.7%)	6 (4.5%)
	Skin, muscles, bones	98	78 (7.3%)	17 (3.4%)	3 (2.2%)
	Unknown without bacteremia	75	46 (4.3%)	26 (5.1%)	3 (2.2%)
	Viral/systemic	42	31 (2.9%)	11 (2.2%)	0 (0.0%)
	Unknown with bacteremia	23	11 (1.0%)	8 (1.6%)	4 (3.0%)
	Central nervous system	18	6 (0.6%)	7 (1.4%)	5 (3.7%)
	Cardiovascular	11	7 (0.7%)	4 (0.8%)	0 (0.0%)
SIRS	Pulse rate	1314	807 (75.2%)	392 (77.5%)	115 (85.8%)
	Temperature	968	639 (59.6%)	263 (52.0%)	66 (49.3%)
	Respiratory rate	1071	631 (58.8%)	343 (67.8%)	97 (72.4%)
	Leucocyte count	1228	760 (70.8%)	368 (72.7%)	100 (74.6%)
SIRS positive criteria, N	2	815	523 (48.7%)	240 (47.4%)	52 (38.8%)
	3	641	409 (38.1%)	178 (35.2%)	54 (40.3%)
	4	257	141 (13.1%)	88 (17.4%)	28 (20.9%)
Site of organ failure	CNS	333	166 (15.5%)	116 (22.9%)	51 (38.1%)
	Metabolic	226	129 (12.0%)	69 (13.6%)	28 (20.9%)
	Cardiovascular	100	58 (5.4%)	33 (6.5%)	9 (6.7%)
	Respiratory	709	471 (43.9%)	194 (38.3%)	44 (32.8%)
	Renal	106	67 (6.2%)	31 (6.1%)	8 (6.0%)
	Hepatic	55	40 (3.7%)	12 (2.4%)	3 (2.2%)
	Coagulation	209	130 (12.1%)	55 (10.9%)	24 (17.9%)

We found that, in the group of patients with sepsis, 390 (62.8%, 95%CI 58.9–66.6%) arrived without public prehospital transport, 197 (31.7%, 95%CI 28.1–35.5%) were transported by ambulance, and 34 (5.5%, 95%CI 3.8–7.7%) were assisted by MECU. In the severe sepsis patients group, the same percentage 62.8% (95%CI 59.9–65.7%) arrived without public pre-hospital transport, a lower percentage 28.2% (95%CI 25.5–31.0%) were transported by ambulance, and a larger percentage 9.0% (95%CI 7.3–10.8%) were transported by MECU compared to the sepsis group.

**Fig. 1:**
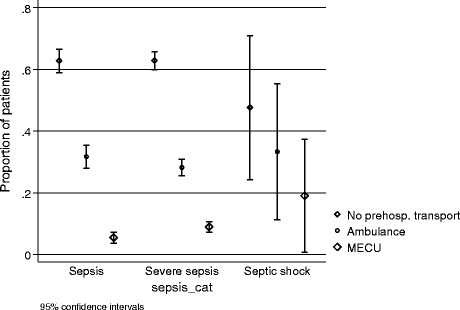
Mode of transport for patients with sepsis, severe sepsis or septic shock, arriving to an Acute Medical Unit

Among 21 patients with septic shock, 10 arrived without public pre-hospital transport (47.7%, 95%CI 59.9–65.7%), 7 (33.3%, 95%CI 14.6–57.0%) were transported by ambulance, and 4 (19.0%, 95%CI 5.4–41.9%) by MECU (Fig. 1).

For all 1,713 patients with sepsis at any severity, the 30-day mortality for patients arriving without public prehospital transport was 12.7%; arriving with ambulance 16% and assisted by MECU 20.9%. In the sepsis group, the 30-day mortality was 6.2, 5.6 and 8.8% in the group with severe sepsis 30-day mortality was 16.2, 23.2, and 22.9% and in the group of septic shock 30-day mortality was 50.0, 12.5, and 37.5%.

The 30-day mortality hazard ratio was associated with mode of transport, with the highest hazard ratio found in the group of MECU transported patients followed by the group of ambulance-transported patients, and the lowest hazard ratio were seen in the patients arriving without public prehospital transport (Table 2).

**Table 2:** Thirty-day mortality and Hazard-Ratio for sepsis patients arriving without public prehospital transport, ambulance or MECU to an Acute Medical Unit

	N	30-day mortality	Crude HR	Adjusted HR (gender and age)	Adjusted HR (all variables)
Gender					
Female	920	132 (14.3%)	1.00 (Ref.)	1.00 (Ref.)	1.00 (Ref.)
Male	793	113 (14.2%)	0.99 (0.77–1.28)	1.02 (0.79–1.31)	0.96 (0.74–1.23)
Age			1.05 (1.04–1.06)	1.05 (1.04–1.06)	1.04 (1.03–1.06)
Charlson Comorbidity Index					
0	570	56 (9.8%)	1.00 (Ref.)	1.00 (Ref.)	1.00 (Ref.)
1–2	415	47 (11.3%)	1.15 (0.78–1.70)	0.76 (0.51–1.12)	0.79 (0.53–1.17)
> 2	728	142 (19.5%)	2.08 (1.52–2.83)	1.23 (0.90–1.68)	1.26 (0.92–1.73)
No. organ dysfunct. per patient					
1	621	38 (6.1%)			
2	651	79 (12.1%)	4.46 (3.01–6.60)	3.34 (2.25–4.96)	3.18 (1.23–8.22)
3+	300	73 (24.3%)	7.14 (4.49–11.34)	5.03 (3.16–8.02)	4.88 (1.90–12.50)
Arrival category					
Without public prehospital transport	1,073	136 (12.7%)	1.00 (Ref.)	1.00 (Ref.)	1.00 (Ref.)
Ambulance	506	81 (16.0%)	1.30 (0.98–1.71)	1.23 (0.93–1.62)	1.30 (0.98–1.71)
MECU	134	28 (20.9%)	1.76 (1.17–2.65)	1.70 (1.13–2.55)	1.76 (1.16–2.66)
Sepsis category					
Sepsis	621	38 (6.1%)	1.00 (Ref.)	1.00 (Ref.)	1.00 (Ref.)
Severe sepsis	1,071	199 (18.6%)	3.30 (2.33–4.66)	2.62 (1.85–3.71)	9.51 (1.96–46.15)
septic shock	21	8 (38.1%)	8.23 (3.84–17.64)	7.39 (3.44–15.87)	8.99 (2.13–38.02)

## Discussion

We found that, among patients with sepsis of any severity, 63% arrived without public prehospital transport, 29% by ambulance, while 8% were assisted by the MECU. We found no clear association between disease severity and mode of transport in the group of patients transported by ambulance, but the ratio of patients transported by MECU increased by disease severity, and the percentage of patients arriving without public prehospital transport decreased by disease severity (Fig. 1). Other studies have described prehospital transport of septic patients in which transportation was classified as Emergency Medical Service (EMS) or non-EMS. In accordance with this study, they found a high percentage of septic patients transported as non-EMS, 49–59% [21–23]. One study of severe septic patients classified 78% as EMS transported patients [24] in contrast to our findings where 37% were transported by Ambulance or MECU. Although, the Danish healthcare system is free, some patients bypass the dispatch system or public prehospital transport, and arrive on their own accord. In contrast to other diseases as trauma [25], cardiac arrest [26, 27], acute myocardial infarction [28] and stroke [29, 30], septic patients, to a lesser extent, arrived by public prehospital transport. In our study, part of the explanation for patients arriving without public pre-hospital transport could be the short distances with low traffic load.

Sepsis has not benefited from the same public focus, as stroke and acute coronary syndromes have [31], and this may explain why these patients are not, to a greater extent, aware of sepsis. Due to the often non-specific presentations of sepsis, it remains challenging. Although screening tools deriving from emergency medical systems data have been developed, these have yet to be incorporated into daily practice [32]. In one study, non-specific diagnosis accounted for one-third of the patients transported by ambulance [33], and the most frequent category were “unclear problems”, when dispatchers assigned a high priority level [1]. One study describing the presentations of sepsis found primarily: Deterioration, physical signs and symptoms and difficulties establishing satisfactory contact with the patient [4]. Septic patients presenting with decreased general condition had less favorable outcome [34], and the risk of having an EMS dispatched as low priority doubled among patients with non-specific complaints [35]. Furthermore patients with decreased general condition in the emergency department, had a four-fold risk of suffering an in-hospital death [36]. To what extent sepsis patients were categorized by the dispatch center as having unclear problems, or presented with decreased general condition, were not part of our study. While symptoms as fever, headache, breathing difficulties, unconsciousness and unclear problem are included in the Danish dispatch system, specific sepsis-related symptoms and descriptions are not [37]. As it is possible that improvement in the very early chain of care in sepsis, treatment could hopefully start even earlier [38]. We hope that future studies including symptom presentation at dispatch, could improve the diagnostic process and facilitate better care of patients with sepsis of any severity. Basis for further research could be case studies on septic patients’, assigned different modes of transport, to increase dispatch-system understanding.

As a secondary aim, we observed a 30-day mortality for patients with sepsis of any severity arriving without public prehospital transport at 12.7%, with ambulance 16% and with MECU 20.9%. Other studies have focused on 28-day mortality and in-hospital mortality for patients with sepsis. These studies reported mortality rates at 15-20% for patients transported by EMS [22, 39, 40] and 6-7% for patients arriving without EMS transport [22, 40]. According to our study and previous studies of patients with sepsis of any severity, mortality changes by mode of arrival. This is most probably caused by the ability of the dispatcher to better discover more serious cases and thus dispatch a suitable resource (MECU and/or ambulance) to these patients. However, we believe there is an even need for greater consistency in the handling of all patients with sepsis, as we found septic patients at any severity at arrival, regardless of transportation mode.

### Study strengths

Due to the uniformly organized Danish public healthcare system, we could identify all patients included in the study. Thus, we present a study with a full medical record including follow up. Manual chart review using a structured protocol was used to collect data regarding the presence of infections and type of transport. Registration of all MECU transports and sampling of paper files identified all ambulance transport. The hospital investigated in this study serves as the primary hospital (and the only hospital) in a well-defined catchment area.

### Limitations

The current work was a single-center study from an acute medical unit at Odense University Hospital. The results may not necessarily be generalized to other hospitals. Moreover, when we compared with studies performed in other countries, the generalizability may be difficult, because of structure differences, differences in which patients have access to the health care system and where the economic resources are focused. Another definition of sepsis would alter the results. Finally there might have been some missed ambulance records, but the number is expected to be limited.

## Conclusions

A substantial proportion of patients with severe sepsis and septic shock arrived to the hospital without public pre-hospital transport, but the proportion of patients with ambulance or MECU transport increased by disease severity. The mortality and hazard-ratio changed by mode of pre-hospital transport, with the highest rate in the MECU-transported patients.
